# Effect of direct surgical treatment in pregnancy-related uterine arteriovenous malformation

**DOI:** 10.1186/s12884-023-05961-3

**Published:** 2023-09-19

**Authors:** Wenjing Zhang, Suhua Cui, Zhonghao Mao, Yiping Hao, Yilin Tan, Yanli Ban, Baoxia Cui

**Affiliations:** 1 Department of Obstetrics and Gynecology, Qilu Hospital of Shandong University, Jinan, Shandong 250012 China; 2 Department of Obstetrics and Gynecology, Qilu Hospital of Shandong University, Qingdao, Shandong 266011 China

**Keywords:** Pregnancy-related UAVM, Treatment method, Uterine artery embolization

## Abstract

**Background:**

Uterine arteriovenous malformation (UAVM) is a relatively rare but potentially life-threatening situations abnormal vascular connections between the uterine arterial and venous systems. Lack of recognized guidelines and clinic experience, there is a lot of clinic problems about diagnosis and treatment. By analyzing the clinical data of patients with pregnancy-related UAVM, we aim to confirm the safety of direct surgeries and the benefit of pretreatment (uterine artery embolization or medical therapy) before surgery, and to explore more optimal therapies for patients with pregnancy-related UAVM.

**Methods:**

A total of 106 patients in Qilu Hospital of Shandong University from January 2011 to December 2021 diagnosed of pregnancy-related UAVM were involved in this study. Depending on whether preoperative intervention was performed, the patients were divided into direct surgery group and pretreatment group (uterine artery embolization or medical management). Clinical characteristics, operative related factors and prognosis were analyzed.

**Results:**

The most common symptom of pregnancy-related UAVM was vaginal bleeding (82.5%), which could also be accompanied by abdominal pain. Pretreatments (uterine artery embolization or medical therapy) had no obvious benefit to the subsequent surgeries, but increased the hospital stay and hospital cost. Direct surgery group had satisfactory success rate and prognosis compared to pretreatment group.

**Conclusion:**

For pregnancy-related UAVM, direct surgery has good effects and high safety with shorter hospital stays and less hospital cost. What is more, without uterine artery embolization and other medical therapy, patients could remain better fertility in future.

**Supplementary Information:**

The online version contains supplementary material available at 10.1186/s12884-023-05961-3.

## Introduction

Uterine arteriovenous malformation (UAVM) is an abnormal vascular connection between the uterine arterial and venous systems, which may lead to severe life-threatening uterine bleeding [1, 2]. UAVM is classified as congenital and acquired UAVM, and the latter includes pregnancy-related and non-pregnancy-related UAVM [2, 3]. Pregnancy-related acquired UAVM is usually secondary to pregnancy-related events, such as villus implantation due to normal position pregnancy and ectopic pregnancy, cesarean section, natural delivery, gestational trophoblastic disease and abortion [2, 4–6]. It was Reported that the prevalence of UAVM was about 4.5% [3], similar to the prospective study from Japan which was about 5.2% of abortions, and 0.22% of delivery [7]. Reported cases are on the rise due to the increasing development of ultrasound, computed tomography (CT), and magnetic resonance imaging (MRI) [8].

Clinical manifestations of acquired UAVMs are mostly menorrhagia, a persistent small amount or a sudden large amount of vaginal bleeding after abortion or uterine curettage. Sometimes, UAVMs might lead to hemodynamic instability, which even endanger life [9, 10], and 30% of patients need blood transfusion [11]. To date, many different treatment options for pregnancy-related UAVM, such as surgical, medical, and uterine artery embolization (UAE), have been applied. However, there is still no consensus on the optimal treatment strategy for UAVM [12]. Therefore, by reviewing different treatment methods and their clinical effect for patients with pregnancy-related UAVM, we hope to confirm the safety of direct surgeries and benefit of UAE or medical therapy before surgery, and explore optimal treatment for pregnancy-related UAVMs patients.

## Methods

A total of 106 patients diagnosed with UAVM were involved in this study. All the patients were admitted to Qilu Hospital of Shandong University from January 2011 to December 2021. All patients underwent an ultrasound examination in our hospital to confirm the diagnosis. Inclusion criteria: (1) ultrasound imaging met the following criteria: uneven echo in the uterine cavity or uterine muscle wall; high peak velocity and low-resistance arterial flows making a “colored mosaic pattern” in CDFI [4, 5]; (2) patient had a pregnancy-related history in the past 60 days including artificial abortion, medical abortion, scar pregnancy, cornual pregnancy, natural delivery and STD. (3) blood HCG test still increased at the time of treatment.Ultrasound diagnosis results of the patients were reviewed and verified by two experienced ultrasound physicians.

All patients underwent surgeries. Depending on whether preoperative intervention was performed, patients were divided into two groups: (1) pretreatment group (33 cases): patients were treated with mifepristone, misoprostol, methotrexate, or UAE before surgery; (2) direct surgery group (73 cases): patients received surgery without any pre-treatments. Operative time, intraoperative blood loss, hospitalization time, hospitalization cost, and prognosis were analyzed between these two groups.

We retrieved ultrasound images of each patient from the ultrasound database to obtain the lesion location, size, blood flow velocity, resistance index (RI) and typical image features. Characteristics and treatments of each patient were obtained from the electronic medical record system, including age, history of present illness, marital and childbearing history, clinical manifestation, inducement, treatment, surgical method, hospitalization time and hospitalization cost. Successful surgical treatment was defined as treatment without hysterectomy and any second surgery. We analyzed the main symptom for analyzing patients’ symptoms. More than twice the amount of menstruation was considered as excessive vaginal bleeding. If one patient had excessive vaginal bleeding and lower abdominal pain, we analyzing excessive vaginal bleeding as her symptoms. What is more, we followed up the patients’ prognosis through telephone, such as menstrual recovery, second pregnancy outcomes, and recurrence. The follow-up ended on November 2022 and lasted from 11 to 130 months.

### Statistics

Statistical software IBM SPSS (ver.23.0) was used. Continuous variables, such as lesion size, were represented as $\stackrel{-}{\text{x}}$±s; while categorical variables, such as age, reproductive history, uterine surgery history, etiology and clinical manifestations, were represented as frequency and percentage. The comparison of basic information (age, reproductive history, clinical manifestations, lesion size, etc.) between the two groups was performed using two independent sample nonparametric tests (U test). *P* value < 0.05 was considered statistically significant.

## Results

### Clinical characteristics

**Fig. 1:**
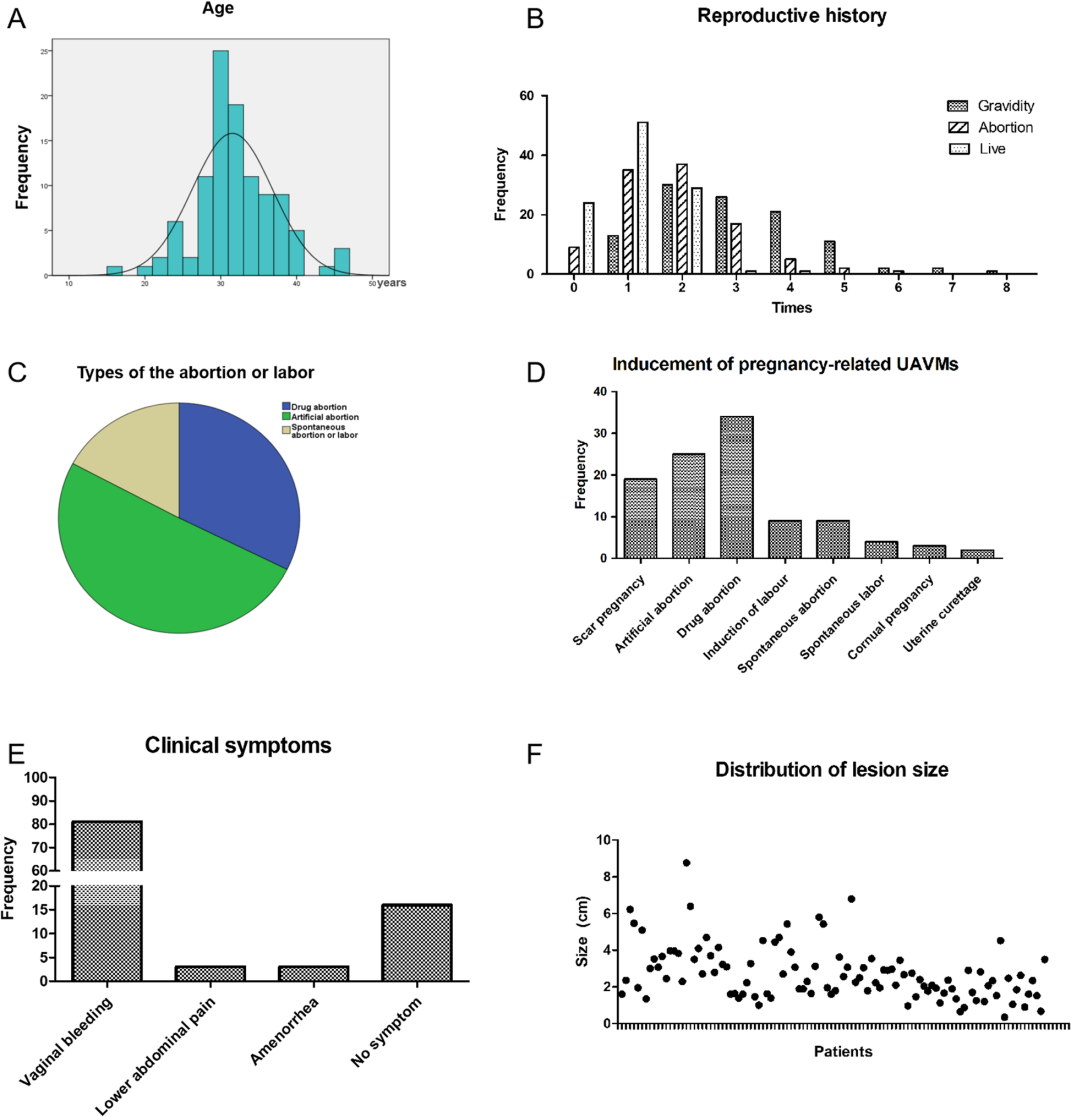
Clinical characters of patients with pregnancy-related UAVMs. **A** The distribution of patients age. **B** Reproductive history of patients. It show the gestation, abortion, and partus maturus. **C** Pregnancy inducement derived into drug abortion, artificial abortion, spontaneous labor and abortion. **D** Inducement of pregnancy-related UAVM for last pregnancy type. **E** Clinical symptoms of all the patients. **F** Lesion size of each patients

**Fig. 2:**
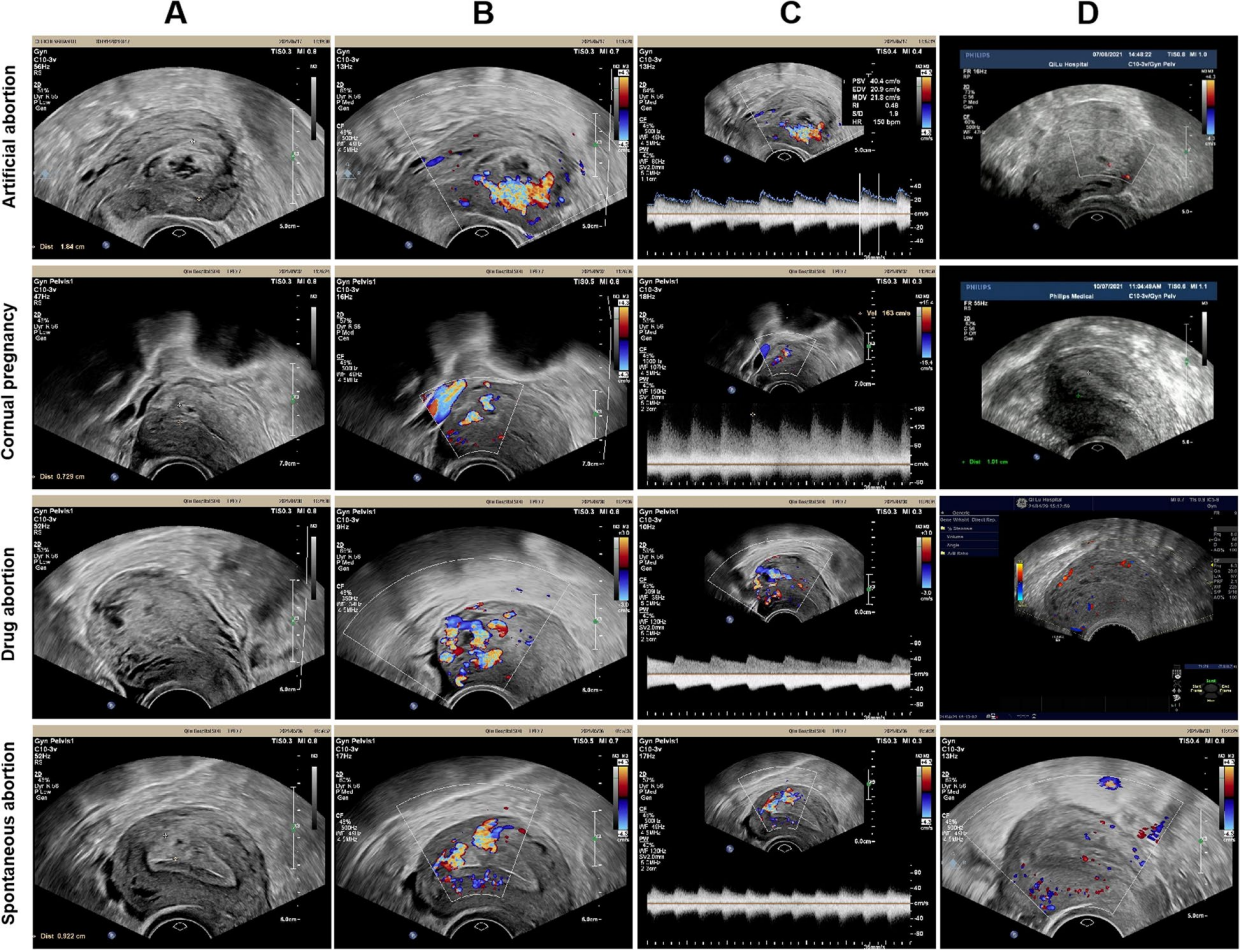
Ultrasound images of patients with pregnancy-related UAVMs. This figure show four patients with pregnancy-related UAVMs. Their related disease are artificial abortion, cornual pregnancy, drug abortion and spontaneous abortion. **A** show the uterine cavity and the UAVM lesion. **B** show the blood flow of the UAVM lesion. **C** show the spectrogram of the UAVM lesion. **D** show the situation after surgery

Of 106 patients, 19 cases were induced after scar pregnancy abortion (5 cases underwent artificial abortion; 14 cases underwent medical abortion), 59 cases had a normal early pregnancy abortion (25 cases underwent artificial abortion; 34 cases underwent medical abortion), 9 cases were induced after spontaneous abortion, 4 cases occurred after spontaneous labor, and 3 cases occurred after cornual pregnancy abortion. The most common clinical symptoms were vaginal bleeding (81 cases), including sudden massive bleeding in 7 cases (1 case underwent induced labor, 1 case underwent an artificial abortion, 1 case underwent a spontaneous labor, 2 cases underwent spontaneous abortion, and 2 cases underwent uterine curettage of scar pregnancy), abdominal pain in 3 cases, amenorrhea in 3 cases. Totally, 16 cases had no obvious clinical manifestations, which only could be detected during the ultrasound examination. All the clinical characteristics are shown in Table 1; Fig. 1. The pre-operation and post-operation (one month after the operation) ultrasonic pictures of several pregnancy-related UAVM cases of direct operation group are shown in Fig. 2.

**Table 1:** Clinical characters of patients with pregnancy-related UAVM

Patients characteristics	Case number
**Age (year)**	
<35	79(74.53%)
≥35	27(25.47%)
**Reproductive history**	
Number of pregnancy	
<3	43(40.57%)
≥3	63(59.43%)
Number of bearing birth	
0	24(22.64%)
1	53(50.00%)
2	27(25.47%)
≥3	2(1.89%)
Number of abortion	
0	9(8.49%)
1	35(32.02%)
2	37(34.91%)
≥3	25(23.58%)
**Operation History**	
Caesarean section	63(59.43%)
Artificial abortion	55(51.89%)
**Inducement**	
Scar pregnancy	19(17.92%)
Artificial abortion	25(23.58%)
Drug abortion	35(33.02%)
Induction of labor	9(8.49%)
Spontaneous abortion	9(8.49%)
Spontaneous labor	4(3.77%)
Cornual pregnancy	3(2.83%)
uterine curettage	2(1.89%)
**Lesion size (cm)**	
≤3	57(53.77%)
>3	49(46.23%)
**Clinical symptoms**	
Vaginal bleeding	83(78.30%)
Lower abdominal pain	4(3.77%)
Amenorrhea	3(2.83%)
No symptom	16(15.09%)

### The overall success rate of each group

In this study, the overall success rate of surgery therapy is 98.1%, 104 out of 106. Among the 106 patients, 2 patients underwent abdominal hysterectomy. The success rate of the direct surgery group is 97.2% (71/73), meanwhile, the pretreatment group is 100% (33/33).

Two patients of direct surgery group underwent hysterectomy, which resulted in failure rate of 2.7% (2/73) in direct surgery group. Case#2 had uterine angle pregnancy with a lesion size of 4.4 cm and rich and tortuous parauterine vessels, who was 36 years old and had no fertility requirement, underwenting abdominal hysterectomy with intraoperative blood loss of 100 mL. Case#5 had a lesion of 4.5 cm which showed a pipe-like echo meaning rich and bulky blood vessels, underwenting abdominal hysterectomy with intraoperative blood loss of 300 mL.

### Intraoperative situations of each group

There was no significant difference in basic characteristics (age, pregnancy, delivery, history of abortion, history of cesarean section, distribution of clinical manifestations, size of lesions, and blood HCG test) between pretreatment group and direct surgery group (*p >* 0.05).

Among the 106 patients, 7 cases underwent uterine curettage, 77 cases underwent hysteroscopy, and 20 cases underwent laparotomy or laporotomy. Most patients had reasonable blood loss, which was less than 400mL. Only 8 patients had more than 400 mL blood loss (one case underwent uterine curettage, 2 cases underwent hysteroscopy, 3 cases underwent laparoscopy, and 2 cases underwent laparotomy).

No significant difference was found in intraoperative blood loss (*p* = 0.106) and operative time (*p* = 0.088), shown in Tables 2 and 3. Compared with the direct surgery group, the pretreatment group had a longer operation time, postoperative hospitalization time and hospitalization time (*p <* 0.001, shown in Table 4).

**Table 2:** Clinic factors between direct surgery group and pretreatment group

Clinic factor	Pretreatment group (*n* = 33)	Direct surgery group (*n* = 73)	*p*
**Age (year)**	32.61	31.07	0.169
**Reproductive history**			
Number of pregnancy	3(2,4)	3(2,4)	0.590
Number of bearing birth	1(1,1)	1(1,2)	0.664
Number of abortion	2(1,2)	2(1,2)	0.277
**Operation History**			
Caesarean section	1(0,1)	0(0,1)	0.105
**Lesion size (cm)**	3.16 ± 0.28	2.53 ± 0.15	0.071
**Clinical symptoms**			
Vaginal bleeding	27(81.82%)	55(75.34%)	-
Lower abdominal pain	2(6.06%)	2(2.74%)	-
No symptom	4(12.12%)	16(21.92%)	-
**HCG (µl/mL)**	89.3(19.82,708.6)	39.33(6.90,474.7)	0.329

**Table 3:** Therapy-related factors between direct surgery group and pretreatment group

Clinic factor	Pretreatment group (*n* = 33)	Direct surgery group (*n* = 73)	*p*
Blood loss in operation (mL)	25(10,100)	20(10,50)	0.106
Operation time (min)	35(21.25,70)	35(25,60)	0.088
Hospital time (day)	10(5,23)	4(3,8)	0.000
Hospital cost (yuan)	21543.70	16247.40	0.043
Operation success ratio	100%	97.3%	-
Reproductive prognosis			
Menstruation resumption	33	70^a^	-
Pregnancy	5	2	-
Not pregnancy	27	71	-
Contraception	25	67	-
No contraception	2	4	-

^a^Two patients had not menstruation of which two had taken hysterectomy, and the other one was in lactation period

**Table 4:** Therapy-related factor between different surgery groups

Operational factor	Uterine curettage (*n* = 8)	Hysteroscopic surgery (*n* = 78)	Laparoscopic/ Abdominal surgery (*n* = 20)	*p*
Blood loss in operation (mL)	30(20,65)	20(10,45)	100(125,350)	*p* < 0.01
Operation time (min)	30(25,70)	30(20,45)	95(73.75,125)	*p* < 0.01
Postoperative hospital stay (day)	6(2,8.5)	2(1.75,3)	7(5.5,8.25)	*p* < 0.01
Reproductive prognosis				
Menstruation resumption	8	77^a^	18^b^	
Pregnancy	2	4	1	
Not pregnancy	6	74	19	
Contraception	4	72	17	
No contraception	1	2	3^b^	

^a^One patient was lactation and had not resumed menstruation

^b^Two patients had taken hysterectomy, did not have menstruation and the chance of pregnancy

### Prognosis of different surgical methods

All patients were asked to take ultrasound examination one month later. Abnormal uterine blood flow signals disappeared or reduced significantly for most patients. Some patients who still had a few abnormal uterine blood flow signals were recommended to repeat ultrasound examination 3 months later, and all of them subsequently restore to normal. The prognosis of different surgical methods is shown in Table 4.

Most patients resumed menstruation at about 4 weeks after surgery. Only 3 patients did not have menstrual recovery at the expected time. One was in lactation period, the other two patients had hysterectomy as mentioned previously. There was no statistical difference on menstrual recovery among different surgery groups.

Eleven patients had fertility requirements, and 7 patients succeeded in pregnancy subsequently, six delivered live infant, while one had a spontaneous miscarriage who underwent partial hysterectomy during previous pregnancy-related UAVMs treatment.

### Special cases and treatments

In this study, a total of 7 cases were suffering excessive vaginal bleeding. Two cases (Case#31 and Case#42) had shock symptoms. For Case#31, massive hemorrhage occurred after artificial abortion with a serum HCG level of 9.48mIU/mL, but stopped after hospitalization. After correcting the condition of hypovolemia, we performed uterine curettage with ultrasonic surveillance, which took 15 min with a blood loss of 30 mL. For Case#42, excessive hemorrhage occurred after induced labor with a serum HCG level of 3.51mIU/mL. She then received hysteroscopic electrotomy after transfusion of 2U RBC, which took 35 min with a blood loss of 50 mL.

The other 5 cases had massive vaginal bleeding without shock symptoms. Two patients (Case#1 and Case#27) with scar pregnancy were hospitalized due to massive vaginal bleeding after induced abortion, who received pretreatment before surgery (Case#1: UAE; Case#27: UAE + MTX 50 mg im). The other 3 cases underwent hysteroscopic electrotomy, and the inducement was spontaneous abortion (Case#5 and Case#102) and spontaneous labor (Case#49).

The level of serum HCG was not high for the patients with excessive vaginal bleeding except the two scar pregnancy patients (Case#1: 1020 mIU/mL; Case#27: 29,256 mIU/mL). Notably, 19 cases complicated with scar pregnancy achieved good outcomes by surgical treatment, according to “Clinical Classification of Cesarean Scar Ectopic Pregnancy” [13, 14].

## Discussion

Pregnancy-related UAVM was over-diagnosed in the past decade [12]. It is more common in women of childbearing age, and most of the patients might have the desire to give birth. Pregnancy-related UAVM may cause vaginal hemorrhage and even life-threatening conditions, and appropriate treatment was the key of favourable prognosis. However, advised medical or surgical management for pregnancy-related UAVMs was predominantly based on UAE [15, 16], which has been proven to be associated with premature ovarian failure and infertility [17]. Therefore, it is very important to adopt an appropriate treatment option to achieve success, protect patients’ fertility and lessen the treatment burden with the least invasive treatment.

In our study, the overall success rate for all patients with surgery treatment is 98.1% (104/106), which is higher than that of medical management (such as progestin, GnRH-a, MTX) reviewed as 88% (106 out of 121 patients) [18]. There is no significant difference of success rate between the direct surgery group (97.2%, 71/73) and the pretreatment group (100%, 33/33). Intraoperative blood loss did not significantly increase for patients in direct surgery group, compared with those who had pretreatment of mifepristone, misoprostol, MTX or UAE before surgery. This result may be explained that UAVM relapse after UAE and other pretreatments could increase the risk of intraoperative bleeding and the difficulty of surgery [18, 19]. However, hospitalization time and hospitalization cost in direct surgery group decreased significantly than those in the pretreatment group. One could argue that the lesion size of pretreatment group is larger than that of the direct surgery group (*p* = 0.071), although the difference was not statistically significant. In fact, there are 18 cases with lesion size over 3 cm (18/33, 54.5%) in pretreatment group, whereas there are 37 cases in direct surgery group (33/73, 45.2%). In addition, for patients in pretreatment group, the lesion size, measured on the day before surgery, might continue to expand after long time of pretreatment. For patients who underwent UAE, it is reasonable to be larger due to more severe vaginal bleeding. All the above results indicated that direct surgery could be recommended as the preferred treatment for pregnancy-related UAVM. Increasing evidence suggests that D&C and operative hysteroscopy without UAE are safe treatment options for pregnancy-related UAVM patients with stable hemodynamics [20–22].

Surgical approaches for pregnancy-related UAVM should be tailored to the ultrasound evaluation of the UAVM lesion (size, depth, PSV) and the patient’s condition. Patients with pregnancy-related UAVM usually have pregnancy residue from previous pregnancies. Most pregnancy-related UAVM lesions are localized, totally in the uterine cavity or partly in the myometrium near to the endometrium. Compared with uterine curettage, hysteroscopy is a better choice to remove the residue and the UAVM lesions located in superficial muscle precisely and to avoid damaging the endometrium at the same time [23]. In order to reduce bleeding and ensure surgical vision, it is recommended to use diluted pituitrin to temporarily limit the local blood supply. At the beginning of hysteroscopic surgery, we injected diluted pituitrin (dilute 6U of pituitrin into 20 mL of 0.9% sodium chloride liquid) into the cervix at 3 and 9 o’clock, 5 ~ 10mL for each site. As pituitrin may cause bradycardia and hypotension, communication with the anesthesiologist should be done before injection (The hysteroscopic surgery video is present in supplementary file 1).

For those lesions of large size and deeply penetrated into the muscle layer or periuterine tissue are not suitable for hysteroscopy. Several other surgical options are available, such as hysteroscopic lesion resection with laparoscopic surveillance or laparoscopic/abdominal lesion resection. In this study, there were totally 20 patients who received laparoscopic/abdominal surgery, pretreatment group occupied 8 (8/37, 21.6%), and direct surgery group occupied 12 (12/73, 16.44%). Moreover, we found that abnormal blood flow in the myometrium faded away naturally after the removal of pregnancy residue. UAVM that persisted in the muscular layer with no symptoms, could be followed up for observation. In patients with shock symptoms of vaginal bleeding UAE may be performed to control hemodynamic stability.

In our study, some patients took DSA, MRI or MRA examination in addition to ultrasound, to confirm the diagnosis. At present, digital subtraction angiography (DSA) is the gold standard for definitive diagnosis of UAVM [8, 24]. Although DSA can complete both diagnosis and embolization at the same time, the practicability of DSA is not high only on the perspective of diagnosis. Furthermore, local arteriovenous fistulas in myometrium are usually very small, can not be presented well by DSA. In our study, ultrasound presentation tended to be a satisfying diagnosis and evaluation method with the advantages of easy, fast, cheap and non-invasive. This was agreed with previous studies [16, 25, 26]. What is more, we preferred to distinguishing pregnancy-related UAVMs from other UAVMs by pregnancy-related history for a short time in the past. However, DSA could be reserved for cases in which surgical intervention or therapeutic embolization of the lesion is planned due to persistent and severe uterine hemorrhage.

There were also several limitations in this current study. First, as this is a retrospective study, there was some bias in the condition of the two groups. A prospective randomized controlled study in the future are suggested. Second, due to the relatively low fertility requirement and small number of patients succeeded in pregnancy subsequently, it is difficult to evaluate the effect of pretreatment on patients’fertility. Longer-term follow-up are considered by us to evaluate the outcomes of pretreatment approaches.

In conclusion, for pregnancy-related UAVM, the key to treatment was to remove intrauterine residual lesions in the conditions of persistent positive HCG and vaginal bleeding. Based on the evaluation of clinical condition, appropriate direct surgery could be considered as the first-line treatment option, among which hysteroscopic resection was recommended as the preferred treatment for pregnancy-related UAVM. However, pretreatment with medical therapy or UAE could not significantly improve the benefit of patients, consequently not recommended as the first choice.

### Supplementary Information

**Additional file 1.**

**Additional file 2.**

## Data Availability

All data generated or analyzed during this study are included in this published article and its supplementary information files.
